# New Staphylinidae (Coleoptera) records with new collection data from New Brunswick, Canada: Omaliinae, Micropeplinae, Phloeocharinae, Olisthaerinae, and Habrocerinae

**DOI:** 10.3897/zookeys.186.2495

**Published:** 2012-04-26

**Authors:** Reginald P. Webster, Jon D. Sweeney, Ian DeMerchant

**Affiliations:** 1 Natural Resources Canada, Canadian Forest Service - Atlantic Forestry Centre, 1350 Regent St., P.O. Box 4000, Fredericton, NB, Canada E3B 5P7

**Keywords:** Staphylinidae, Omaliinae, Micropeplinae, Phloeocharinae, Olisthaerinae, Habrocerinae, new records, Canada, New Brunswick

## Abstract

Eleven species of Omaliinae are newly recorded from New Brunswick, bringing the total number of species known from the province to 32 described species. Supporting data are presented for the New Brunswick record of *Geodromicus strictus* (Fauvel) reported by Majka et al. (2011). *Micropeplus browni* Campbell, *Micropeplus laticollis* Mäklin (Micropeplinae), *Charyhyphus picipennis* (LeConte) (Phloeocharinae*), Olisthaerus substriatus* (Paykull) (Olisthaerinae), *Habrocerus capillaricornis* (Gravenhorst), *Habrocerus magnus* LeConte, and *Habrocerus schwarzi* Horn (Habrocerinae) are also newly recorded for New Brunswick. These are the first records of the latter four subfamilies from New Brunswick. Collection and bionomic data are presented for each species and discussed.

## Introduction

This paper treats new records from New Brunswick of the family Staphylinidae from the subfamilies Omaliinae, Micropeplinae, Phloeocharinae, Olisthaerinae, and Habrocerinae. Most genera of Omaliinae occurring in eastern Canada are relatively well known taxonomically as a result of revisions by Campbell (1978a) (*Boreaphilus* and *Coryphium*), Campbell (1982) (*Acidota*), Campbell (1983a) and Gusarov (1995) (*Pycnoglypta*), Campbell (1983b) (*Olophrum*), Campbell (1984a) (*Arpedium* and *Eucnecosum*), Campbell (1984b) (*Porrhodites*), and Smetana (1996) (*Trigonodemus*). However, the *Omalium* and *Phyllodrep*a need revision as there are several undescribed species, including an undescribed *Omalium* sp. that is known from New Brunswick and other areas in eastern Canada.

The Omaliinae occur in a variety of habitats and can be found in various kinds of decaying organic material, in fungi, on flowers (*Eusphalerum*), and in various wetland habitats, such as marshes, bogs, and various riparian habitats. *Micralymma marinum* (Ström) is intertidal and probably feeds on various arthropods living in this habitat, including the intertidal collembolan *Anurida maritima* (Guérin) (Thayer 1985). Details on habitat associations and biology of the various genera of Omaliinae are included in the revisions above and in various references cited in Newton et al. (2000).

Seventeen species of Omaliinae were reported from New Brunswick by Campbell and Davies (1991). Three additional species (*Omalium foraminosum* Mäklin, *Omalium quadripenne* Casey, *Omalium rivulare* (Paykull)) were reported from the province by Klimaszewski et al. (2005). Majka et al. (2011) reported *Geodromicus strictus* (Fauvel) as occurring in New Brunswick but did not provide any supporting data for the record. Here, we report another 11 species of Omalinae from New Brunswick, including supporting data for *Geodromicus strictus*.

A brief synopsis of the subfamilies Micropeplinae, Phloeocharinae, Olisthaerinae, and Habrocerinae is presented with the respective species accounts below.

## Methods and conventions

The following records are based on specimens collected as part of a general survey by the first author to document the Coleoptera fauna of New Brunswick and from by-catch samples from Lindgren 12-funnel traps (Lindgren 1983) obtained during a study to develop a general attractant for the detection of invasive species of Cerambycidae.

### Collection methods

Various collection methods were employed to collect the species reported in this study. Details are outlined in Campbell (1973a) and Webster et al. (2009, Appendix). See Webster et al. (2012) for details of the methods used to deploy Lindgren funnel traps and for sample collection. A description of the habitat was recorded for all specimens collected during this survey. Locality and habitat data are presented exactly as recorded on labels for each specimen. This information, as well as additional collecting notes, is summarized and discussed in collection and habitat data for each species.

### Specimen preparation

Examples of males of some species were dissected to confirm their identity. The genital structures were dehydrated in absolute alcohol, mounted in Canada balsam on celluloid microslides, and pinned with the specimens from which they originated.

### Distribution

Distribution maps, created using ArcMap and ArcGIS, are presented for each species in New Brunswick. Every species is cited with current distribution in Canada and Alaska, using abbreviations for the state, provinces, and territories. New provincial records are indicated in bold under Distribution in Canada and Alaska. The following abbreviations are used in the text:

**Table:** 

**AK**	Alaska	**MB**	Manitoba
**YT**	Yukon Territory	**ON**	Ontario
**NT**	Northwest Territories	**QC**	Quebec
**NU**	Nunavut	**NB**	New Brunswick
**BC**	British Columbia	**PE**	Prince Edward Island
**AB**	Alberta	**NS**	Nova Scotia
**SK**	Saskatchewan	**NF & LB**	Newfoundland and Labrador*

*Newfoundland and Labrador are each treated separately under the current Distribution in Canada and Alaska.

Acronyms of collections examined and referred to in this study are as follows:

**AFC** Atlantic Forestry Centre, Natural Resources Canada, Canadian Forest Service, Fredericton, New Brunswick, Canada

**CNC** Canadian National Collection of Insects, Arachnids and Nematodes, Agriculture and Agri-Food Canada, Ottawa, Ontario, Canada

**NBM** New Brunswick Museum, Saint John, New Brunswick, Canada

**RWC** Reginald P. Webster Collection, Charters Settlement, New Brunswick, Canada

## Results

Eleven species of Omaliinae are newly recorded from New Brunswick, bringing the total number of species known from the province to 33. Five of the 11 species are also newly recorded for the Maritime provinces (New Brunswick, Nova Scotia, Prince Edward Island). *Micropeplus browni* Campbell, *Micropeplus laticollis* Mäklin (Micropeplinae), *Charyhyphus picipennis* (LeConte) (Phloeocharinae*), Olisthaerus substriatus* (Paykull) (Olisthaerinae), *Habrocerus capillaricornis* (Gravenhorst), *Habrocerus magnus* LeConte, and *Habrocerus schwarzi* Horn (Habrocerinae) represent the first records of these species and four subfamilies for New Brunswick. A list of species of Omaliinae, Micropeplinae, Phloeocharinae, Olisthaerinae, and Habrocerinae known from New Brunswick is presented in Table 1.

**Table 1.** Species of Omaliinae, Micropeplinae, Phloeocharinae, Olisthaerinae, and Habrocerinae known from New Brunswick, Canada.<br/>

**Family Staphylinidae Latreille**
**Subfamily Omaliinae MacLeay**
**Tribe Omaliini MacLeay**
*Acrolocha diffusa* (Fauvel)
*Hapalaraea hamata* (Fauvel)*
*Micralymma marinum* (Ström)
*Omalium foraminosum* Mäklin
*Omalium quadripenne* Casey
*Omalium rivulare* (Paykull)
*Omalium* (undescribed species)
*Phloeonomus laesicollis* (Mäklin)*
*Phloeostiba lapponica* (Zetterstedt)
*Pycnoglypta aptera* Campbell
*Pycnoglypta campbelli* Gusarov
**Tribe Eusphalerini Hatch**
*Eusphalerum convexu*m (Fauvel)
*Eusphalerum fenyesi* (Bernhauer)
*Eusphalerum pothos* (Mannerheim)
**Tribe Anthophagini Thomson**
*Acidota crenata* (Fabricius)
*Acidota quadrata* (Zetterstedt)**
*Acidota subcarinata* Erichson
*Arpedium angulare* Fauvel
*Eucnecosum brunnescens* (J. Sahlberg)**
*Eucnecosum tenue* (LeConte)**
*Brathinus nitidus* LeConte
*Brathinus varicornis* LeConte
*Geodromicus plagiatus* Say
*Geodromicus strictu*s (Fauvel)*
*Lesteva pallipes* LeConte
*Microedus austinianus* LeConte*
*Olophrum consimile* (Gyllenhal)
*Olophrum obtectum* Erichson**
*Olophrum rotundicolle* (C.R. Sahlberg)*
*Porrhodites inflatus* (Hatch)**
*Trigonodemus striatus* LeConte*
**Tribe Coryphiini Jakobson**
*Coryphium nigrum* Campbell*
*Boreaphilus henningianus* C.R. Sahlberg
**Subfamily Micropeplinae Leach**
*Micropeplus browni* Campbell**
*Micropeplus laticollis* Mäklin**
**Subfamily Phloeocharinae Erichson**
*Charhyphus picipennis* (LeConte)*
**Subfamily Olisthaerinae Thomson**
*Olisthaerus substriatus* (Paykull)
**Subfamily Habrocerinae Mulsant & Rey**
*Habrocerus capillaricornis* (Gravenhorst)*
*Habrocerus magnus* LeConte**
*Habrocerus schwarzi* Horn**

**Notes:** *New to province, **New to Maritime provinces.

## Species accounts

All records below are species newly recorded for New Brunswick, Canada. Species followed by ** are newly recorded from the Maritime provinces.

The suprageneric classification of the Omaliinae, Micropeplinae, Phloeocharinae, Olisthaerinae, and Habrocerinae follows Bouchard et al. (2011).

### Family Staphylinidae Latreille, 1802. Subfamily Omaliinae MacLeay, 1825. Tribe Omaliini, MacLeay, 1825

#### Hapalaraea hamata

(Fauvel, 1878)

http://species-id.net/wiki/Hapalaraea_hamata

Map 1

##### Material examined.

**New Brunswick, Carleton Co.**, Jackson Falls, Bell Forest, 46.2200°N, 67.7231°W, 19–27.VI.2008, 19–28.VII.2008, R. P. Webster, mature hardwood forest with some conifers, Lindgren funnel traps (3 ♂, 1 sex undetermined, AFC, RWC). **Gloucester Co.**, Jacquet River Gorge P.N.A. (Protected Natural Area), 47.7684°N, 65.9396°W, 7–18.VIII.2010, K. Vandenbroek & A. Fairweather, northern hardwood forest near Big Meadow, Lindgren funnel trap (1, NBM). **Queens Co.**, Pleasant Villa, 45.7023°N, 66.1732°W, 15.VI.2007, S. Makepeace & R. Webster, nest contents of barred owl (1 ♂, 2 ♀, NBM, RWC); Central Hampstead, 45.6575°N, 66.1412°W, 13.VII.2006, Scott Makepeace, hardwood forest, in nest contents of barred owl in tree hole (1 ♂, RWC); Cranberry Lake P.N.A, 46.1125°N, 65.6075°W, 10–15.VII.2009, 21–28.VII.2009, 19.VIII–2.IX.2009, R. Webster & M.-A. Giguère, old red oak forest, Lindgren funnel traps (3, RWC). **Restigouche Co.**, Dionne Brook P.N.A., 47.9030°N, 68.3503°W, 9–23.VIII.2011, M. Roy & V. Webster, old-growth northern hardwood forest, Lindgren funnel trap (1, NBM). **Sunbury Co.**, Noonan, 45.9923°N, 66.4099°W, 22.VI.2007, S. Makepeace & R. Webster, nest contents of barred owl from tree hole 7 m high in red maple, damp organic material with small bones (1 ♂, 2 ♀, 1 sex undetermined, NBM, RWC);Acadia Research Forest, 45.9866°N, 66.3841°W, 13–21.VII.2009, R. Webster & M.-A. Giguère, mature (110 year-old) red spruce forest with scattered red maple and balsam fir, Lindgren funnel trap (1, AFC). **York Co.**, near “Browns Mountain Fen”, 45.8876°N, 67.6560°W, 3.VIII.2006, R. P. Webster, hardwood forest, on gilled mushroom on tree (1 ♂, RWC); 15 km W of Tracy off Rt. 645, 45.6848°N, 66.8821°W, 29.VII–4.VIII.2009, R. Webster & M.-A. Giguère, old red pine forest, Lindgren funnel traps (2, AFC); same locality and habitat data, 20.VI–13.VII.2010, R. Webster & K. Burgess, Lindgren funnel trap (1, AFC).

**Map 1.**
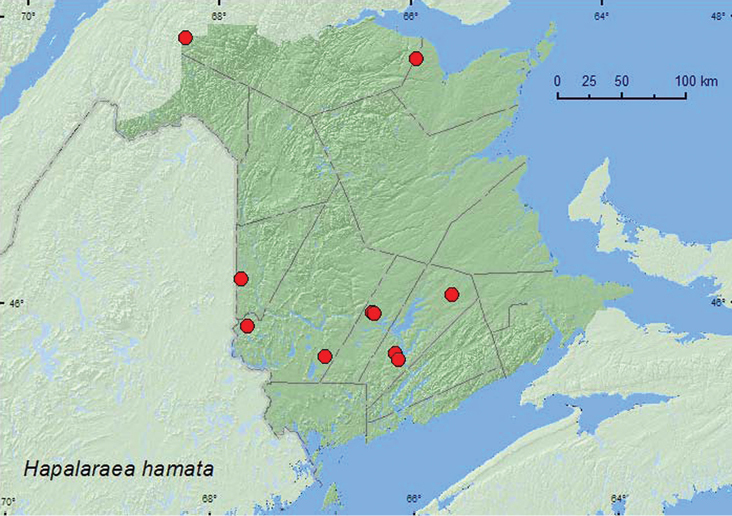
Collection localities in New Brunswick, Canada of *Hapalaraea hamata*.

##### Collection and habitat data.

This species was collected from nest contents of barred owls (*Strix varia* Barton) and from a gilled mushroom on a tree. Adults were also collected from Lindgren funnel traps deployed in hardwood forests with sugar maple (*Acer saccharum* Marsh.) and American beech (*Fagus grandifolia* Ehrh.), an old red oak (*Quercus rubra* L.) forest, an old-growth northern hardwood forest, a 110-year-old red spruce (*Picea rubens* Sarg.) forest, and an old red pine (*Pinus resinosa* Ait.) forest. This species is probably associated with decaying organic materials associated with standing trees. Adults were collected during June, July, August, and September.

##### Distribution in Canada and Alaska.

BC, MB, ON, QC, **NB**, NS (Campbell and Davies 1991; Hammond et al. 2004).

#### Phloeonomus laesicollis

(Mäklin, 1852)

http://species-id.net/wiki/Phloeonomus_laesicollis

Map 2

##### Material examined.

**New Brunswick, Carleton Co.**, Jackson Falls, Bell Forest, 46.2200°N, 67.7231°W, 4–12.VI.2008, R. P. Webster, mature hardwood forest, Lindgren funnel traps (2, RWC). **Restigouche Co.**, Jacquet River Gorge P.N.A., 47.7879°N, 66.0013°W, 13.VI.2009, R. P. Webster, mixed forest, under birch bark (with fermented sap) (1, RWC); Dionne Brook P.N.A., 47.9064°N, 68.3441°W, 31.V–15.VI.2011, 28.VII–9.VIII.2011, M. Roy & V. Webster, old-growth white spruce and balsam fir forest, Lindgren funnel traps (2, AFC, NBM). **York Co.**, Charters Settlement, 45.8395°N, 66.7391°W, 30.V.2007, 5.VI.2007, R. P. Webster, mixed forest, under tight bark on dead standing balsam fir (3, RWC); McAdam, Georgia Pacific Plywood Mill, 19.V.1978, (no collector given) from pile of plywood disks (1, AFC); 15 km W of Tracy off Rt. 645, 45.6848°N, 66.8821°W, 8–15.VI.2009, 14–20.VII.2009, 20–29.VII.2009, 29.VII–4.VIII.2009, 4–11.VIII.2009, R. Webster & M.-A. Giguère, old red pine forest, Lindgren funnel traps (6, AFC, RWC); 14 km WSW of Tracy, S of Rt. 645, 45.6741°N, 66.8661°W, 10–26.V.2010, R. Webster & C. MacKay, old mixed forest with red and white spruce, red and white pine, balsam fir, eastern white cedar, red maple, and *Populus* sp., Lindgren funnel trap (1, AFC).

**Map 2.**
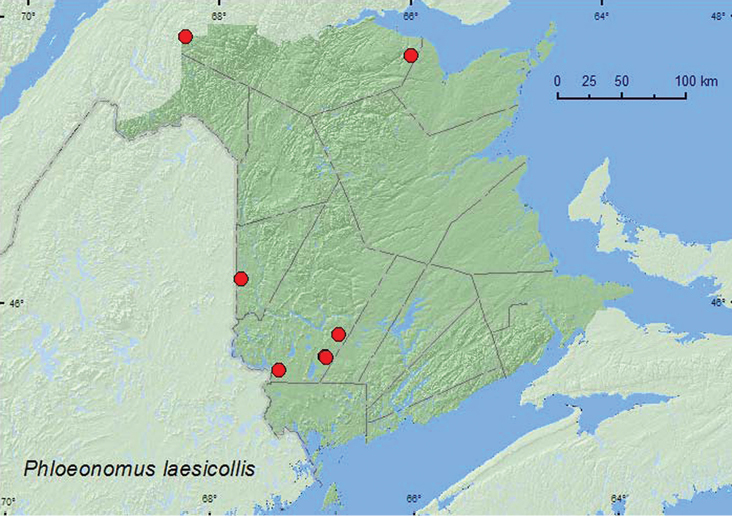
Collection localities in New Brunswick, Canada of *Phloeonomus laesicollis*.

##### Collection and habitat data. 

This species lives under bark of logs and trees (Deyrup and Gara 1979; Newton et al. 2000). In New Brunswick, adults were collected from under tight-fitting bark of a standing dead birch (*Betula* sp.) and a standing dead balsam fir (*Abies balsamea* (L.) Mill.), and from a pile of plywood disks. Adults were captured in Lindgren funnel traps at several sites. Adults were collected during May, June, July, and August.

##### Distribution in Canada and Alaska.

AK, BC, AB, ON, QC, **NB**, NS, NF (Campbell and Davies 1991, as *pusillus* (Gravenhorst)).

### Tribe Anthophagini Thomson, 1859

#### Acidota quadrata

(Zetterstedt, 1838)**

http://species-id.net/wiki/Acidota_quadrata

Map 3

##### Material examined.

**New Brunswick,, Restigouche Co.**, Berry Brook P.N.A (Protected Natural Area), 47.8140°N, 66.7578°W, 26.V.2007, R. P. Webster, old growth eastern white cedar swamp, in moss and leaf litter near brook (1, RWC).

**Map 3.**
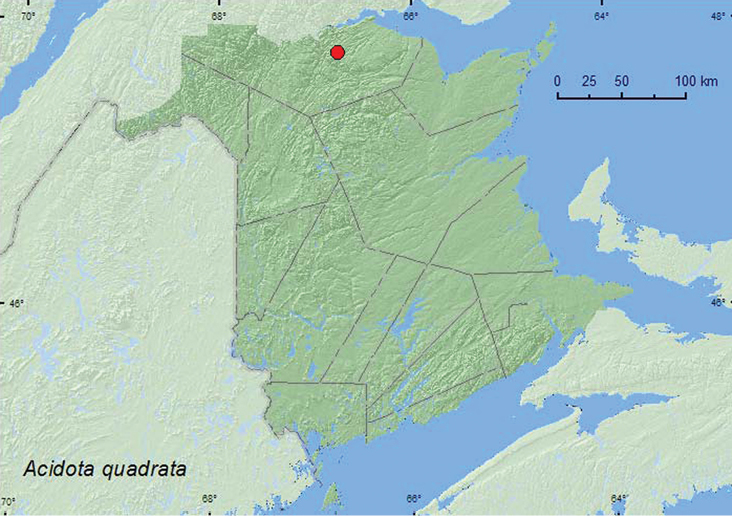
Collection localities in New Brunswick, Canada of *Acidota quadrata*.

##### Collection and habitat data.

*Acidota quadrata* occurs in arctic and alpine tundra areas south into the boreal forest (Campbell 1982). Relict populations from New Hampshire and Maine occur in alpine areas. Adults have been found in leaf litter, in wet moss, and under rocks near streams, in flood debris near rivers, inside a beaver lodge, and in wet moss and clumps of dead grass in alpine tundra (Campbell 1982). The specimen from New Brunswick was sifted from moss and leaf litter, near a brook in an old-growth eastern white cedar (*Thuja occidentalis* L.) swamp during May.

##### Distribution in Canada and Alaska.

AK, YT, NT, BC, AB, MB, ON, QC, **NB**, LB (Campbell 1982). This is a northern Holarctic species known from Alaska to Labrador, south at higher elevations to British Columbia and northern Montana, with relict populations in the mountains of Colorado, New Hampshire, and Maine (Campbell 1982).

#### Eucnecosum brunnescens

(J. Sahlberg, 1871)**

http://species-id.net/wiki/Eucnecosum_brunnescens

Map 4

##### Material examined.

**New Brunswick, Madawaska Co.**, Loon Lake, 236 m elev., 47.7839°N, 68.3943°W, 21.VI.2010, 21.VII.2010, R. P. Webster, boreal forest, small lake surrounded by sedges, treading sedges and grasses near *Myrica gale* bushes into water (9 ♂, 8 ♀, NBM, RWC). **Restigouche Co.**, Jacquet River Gorge P.N.A. 47.8200°N, 66.0015°W, 13.V.2010, R. P. Webster, under alders near brook in *Carex* marsh, in leaf litter and moss (1 ♂, 1 ♀, NBM); same locality but 47.8257°N, 66.0779°W, 24.V.2010, R. P. Webster, partially shaded cobblestone bar near outflow of brook into Jacquet River, under cobblestone in sand gravel mix (1♀, RWC).

**Map 4.**
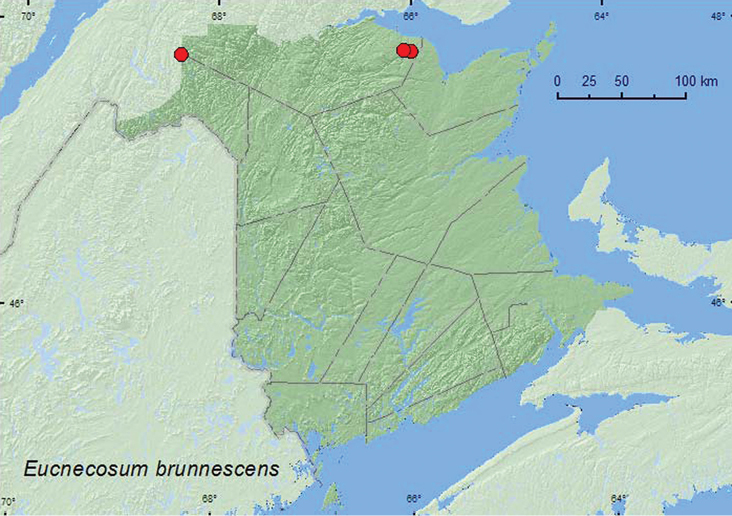
Collection localities in New Brunswick, Canada of *Eucnecosum brunnescens*.

##### Collection and habitat data.

Adults of this northern species are typically found by sifting *Alnus* and *Salix* spp. litter near margins of bogs, shallow lakes, and streams (Campbell 1984a). They are also found in *Carex* hummocks (collected by treading hummocks into water) and in bird nests on ground (Campbell 1984a). Most of the New Brunswick specimens were collected from a wet (emergent sedges) sedge marsh near a small lake by treading sedges and grasses near *Myrica* bushes into water. Two adults were found in leaf litter and moss under alders near a small brook in a *Carex* marsh, and one individual was found under a cobblestone on a shaded cobblestone bar in a brook. Adults were collected during May, June, and July.

##### Distribution in Canada and Alaska.

AK, YT, NT, BC, AB, MB, ON, QC, **NB**, LB, NF (Campbell 1984a). This is a widely distributed Holarctic species found across Canada in the boreal forest areas, north to the southern Arctic (Campbell 1984a).

#### Eucnecosum tenue

(LeConte, 1863)**

http://species-id.net/wiki/Eucnecosum_tenue

Map 5

##### Material examined.

**New Brunswick, Madawaska Co.**, Loon Lake, 236 m elev., 47.7839°N, 68.3943°W, 21.VI.2010, R. P. Webster, boreal forest, small lake surrounded by sedges, treading sedges and grasses into water (2 ♂, RWC).

**Map 5.**
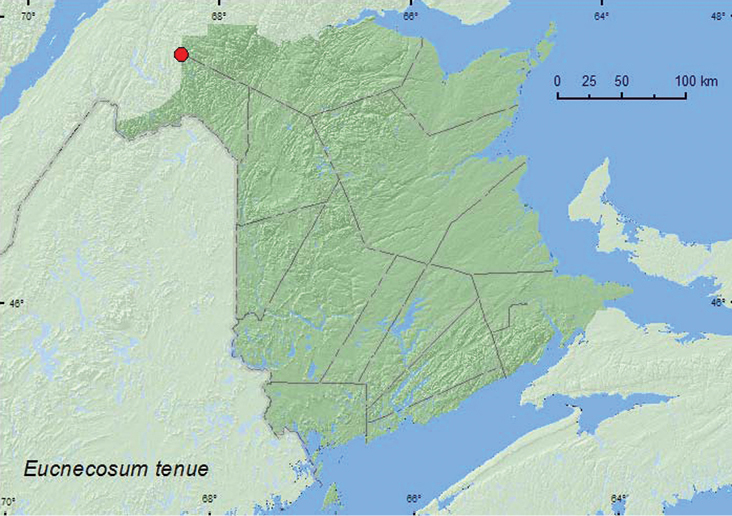
Collection localities in New Brunswick, Canada of *Eucnecosum tenue*.

##### Collection and habitat data.

Adults of this northern species are typically found in *Alnus* and *Salix* spp. litter by sifting and by treading vegetation on margins of bogs, shallow lakes, and streams (Campbell 1984a). The two specimens from New Brunswick were collected from a wet (emergent sedges) sedge marsh near a small lake by treading sedges and grasses into water. The adults were collected during June.

##### Distribution in Canada and Alaska.

AK, YT, NT, BC, AB, SK, MB, ON, QC, **NB**, NF, LB (Campbell 1984a). This is a widely distributed Holarctic species found across Canada in the boreal forest areas north to the southern Arctic (Campbell 1984a).

#### Geodromicus strictus

Fauvel, 1889

http://species-id.net/wiki/Geodromicus_strictus

Map 6

##### Material examined.

**Additional New Brunswick records. Albert Co.**, Caledonia Gorge P.N.A. at Caledonia Creek, 45.7935°N, 64.7760°W, 1.VII.2011, R. P. Webster, shaded, rocky, cold, clear brook, splashing exposed rocks (3, NBM). **Carleton Co.**, Jackson Falls, 46.2257°N, 67.7437°W, 12.IX.2009, R. P. Webster, river margin near waterfalls, splashing moss near splash zone of waterfalls (5 ♂, 6 ♀, NBM, RWC). **Madawaska Co.**, Edmunston, 22.VI.1983, L. LeSage, small creek with bottom of cobbles (1, CNC); Gagné Brook at First Lake, 47.6077°N, 68.2534°W, 23.VI.2010, M. Turgeon & R. Webster, northern hardwood forest, shaded brook among gravel on gravel bar, splashing and turning pebbles (1 ♂, NBM); Jalbert Brook, 262 m elev., 47.6470°N, 68.3026°W, 23.VI.2010, R. P. Webster, old growth mixed forest, shaded brook, in gravel on gravel bar (2 ♂, NBM, RWC). **Northumberland Co.**, Trout Brook, 22.VII.1962, J. Marshall (1, CNC). **Restigouche Co.**, Jacquet River Gorge P.N.A., Jacquet River, 47.8164°N, 66.0873°W, 15.VIII.2010, R. P. Webster, clear rocky fast flowing river, splashing rocks in middle of river (2 ♂, 1 ♀, NBM, RWC); Kedgwick Forks, 47.9085°N, 67.9057°W, 22.VI.2010, R. P. Webster, on exposed rocks in middle of river (2 ♀, NBM, RWC).

**Map 6.**
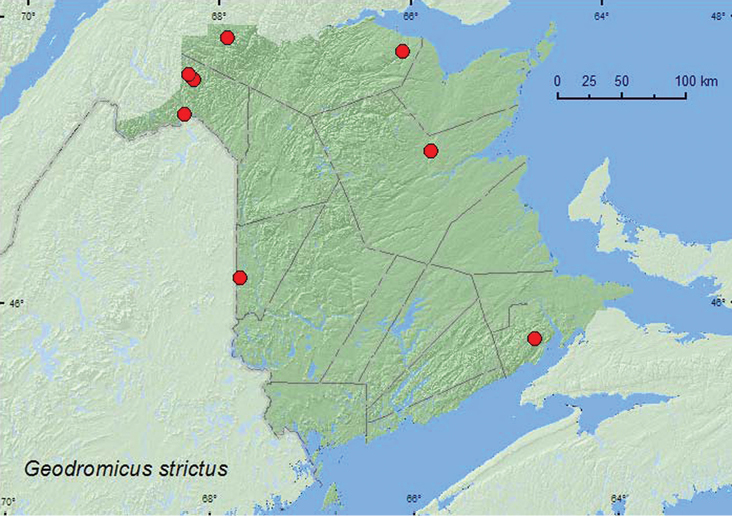
Collection localities in New Brunswick, Canada of *Geodromicus strictus*.

##### Collection and habitat data.

In New Brunswick,*Geodromicus strictus* was usually found on exposed rocks and among cobblestones in the middle of clear, fast-flowing rivers or in moss on rocks adjacent to fast-flowing water near waterfalls. A few adults were collected from cobblestones in shaded brooks. Adults were collected by splashing rocks or cobblestones. This species was collected during June, July, August, and September.

##### Distribution in Canada and Alaska.

ON, QC, **NB**, NS, PE, NF (Campbell and Davies 1991; CNC specimens). *Geodromicus strictus* was listed as occurring in New Brunswick by Majka et al. (2011) without any supporting references or data. Here we provide the first documented records from New Brunswick.

#### Microedus austinianus

LeConte, 1874

http://species-id.net/wiki/Microedus_austinianus

Map 7

##### Material examined.

**New Brunswick, Albert Co.**, Caledonia Gorge P.N.A. at Caledonia Creek, 45.7935°N, 64.7760°W, 1.VII.2011, R. P. Webster, shaded, rocky, cold, clear brook, splashing gravel (4, NBM, RWC). **Madawaska Co.**, Gagné Brook at First Lake, 47.6077°N, 68.2534°W, 23.VI.2010, M. Turgeon & R. Webster, northern hardwood forest, shaded brook among gravel on gravel bar, splashing and turning pebbles (3 ♂, 1 ♀, NBM, RWC); Jalbert Brook, 262 m elev., 47.6470°N, 68.3026°W, 23.VI.2010, R. P. Webster, old growth mixed forest, shaded brook, in gravel on gravel bar (3 ♂, 2 ♀, NBM, RWC). **Restigouche Co.**, Jacquet River Gorge P.N.A., 47.8010°N, 66.0963°W, 14.VI.2009, R. P. Webster, cold shaded brook margin, in fine gravel (1 ♂, 2 ♀, NBM, RWC); 1.5 km S of Quebec border, 47.9058°N, 68.1505°W, 22.VI.2010, R. P. Webster, boreal forest, small cold shaded brook, splashing gravel on gravel bar (6, NBM, RWC); Mount Atkinson, 447 m elev., 47.8192°N, 68.2618°W, 21.VII.2010, R. P. Webster, boreal forest, small (cold spring-fed) shaded brook with mossy margin, in gravel (1, RWC); Kedgwick Forks, 47.9085°N, 67.9057°W, 22.VI.2010, R. P. Webster, on exposed rocks in middle of river (1 ♀, NBM).

**Map 7.**
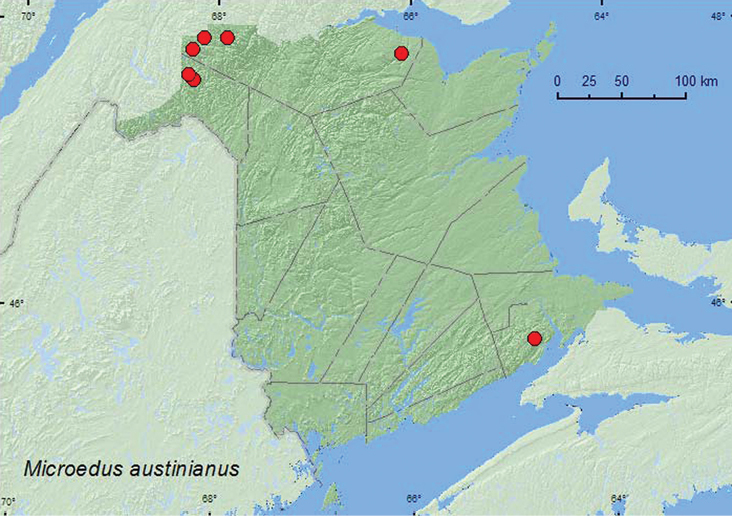
Collection localities in New Brunswick, Canada of *Microedus austinianus*.

##### Collection and habitat data.

In New Brunswick, this species was usually found among gravel on small, shaded, gravel bars or gravel margins of cold shaded brooks. Adults were collected during June and July.

##### Distribution in Canada and Alaska.

AK, YK, BC, AB, QC, **NB**, NS (Campbell and Davies 1991; CNC specimens).

#### Olophrum obtectum

Erichson, 1840**

http://species-id.net/wiki/Olophrum_obtectum

Map 8

##### Material examined. 

**New Brunswick, Queens Co.**, Upper Gagetown, bog adjacent to Hwy 2, 45.8316°N, 66.2346°W, 23.V.2006, R. P. Webster, tamarack bog, treading *Carex* into water (7, RWC). **Saint John Co.**, Musquash, 45.1837°N, 66.3376°W, 7.V.2006, R. P. Webster, inland margin of salt marsh, in litter on muddy soil (1, RWC).

**Map 8.**
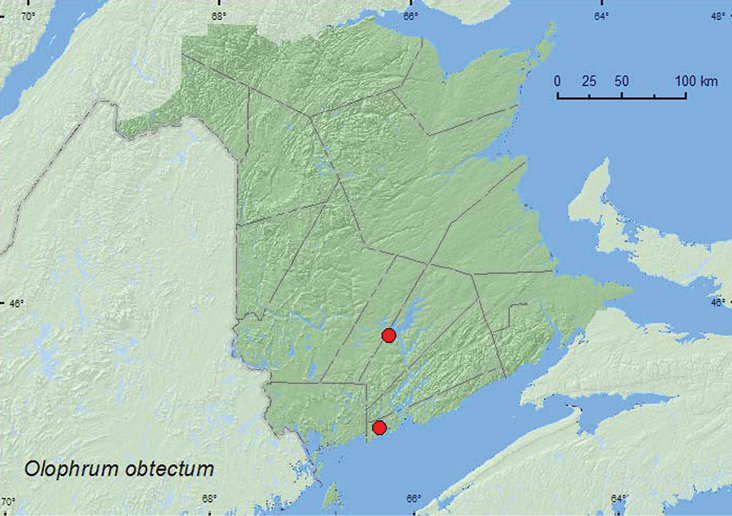
Collection localities in New Brunswick, Canada of *Olophrum obtectum*.

##### Collection and habitat data.

Specimens have been collected from moss along a stream margin, sweeping vegetation along a stream margin, from a Berlese sample from a decayed stump, at light and from emergent *Carex* in an alder swamp (Campbell 1983b). In New Brunswick, adults were common among emergent *Carex* in an open section of a tamarack (*Larix laricina* (Du Roi) Koch) bog near a small, slow-flowing stream. Adults were collected by treading vegetation into water. One adult was also collected from litter (mostly *Carex* sp.) on muddy soil on the inland margin of a salt marsh. All adults were collected during May.

##### Distribution in Canada and Alaska.

ON, QC, **NB** (Campbell 1983b). This species occurs in the eastern third of the United States northward to Quebec and Ontario (Campbell 1983b).

#### Olophrum rotundicolle

(C. R. Sahlberg, 1830)

http://species-id.net/wiki/Olophrum_rotundicolle

Map 9

##### Material examined.

**New Brunswick, Kent Co.**, Kouchibouguac Nat. Park, 20.IX.1978, J, J. Miller (1 ♂, CNC). **Madawaska Co.**, Loon Lake, 236 m elev., 47.7839°N, 68.3943°W, 21.VII.2010, R. P. Webster, boreal forest, small lake surrounded by sedges, treading sedges and grasses near *Myrica gale* bushes into water (2, RWC). **Restigouche Co.**, Little Tobique River near Red Brook, 47.4462°N, 67.0689°W, 13.VI.2006, 24.V.2007, R. P. Webster, old growth eastern white cedar swamp, in moss and leaf litter near brook (1, RWC); MacFarlane Brook P.N.A, 47.6018°N, 67.6263°W, 25.V.2007, R. P. Webster, old growth eastern white cedar swamp, in moss near brook (3, NBM, RWC); Jacquet River Gorge P.N.A. 47.8200°N, 66.0015°W, 13.V.2010, R. P. Webster, under alders near brook in *Carex* marsh, in leaf litter and moss (1 ♀, NBM). **Saint John Co.**, Bains Corner, 45.3223°N, 65.6663°W, 26.V.2006, R. P. Webster, eastern white cedar swamp, in moss (wet) and leaf litter (1, RWC). **Victoria Co.**, 4.0 km NE of Black Brook, 47.3755°N, 67.0100°W, 27.VIII.2004, D. Sabine & R. P. Webster, coll., calcareous eastern white cedar fen, in drainage ditch with sphagnum and sedges (1, RWC). **York Co.** Charters Settlement, 45.8267°N, 66.7343°W, 30.IV.2005, 23.V.2005, 21.V.2007, R. P. Webster, *Carex* marsh, treading *Carex* hummocks with sphagnum into water (1 ♂, 4 sex undetermined, NBM, RWC).

**Map 9.**
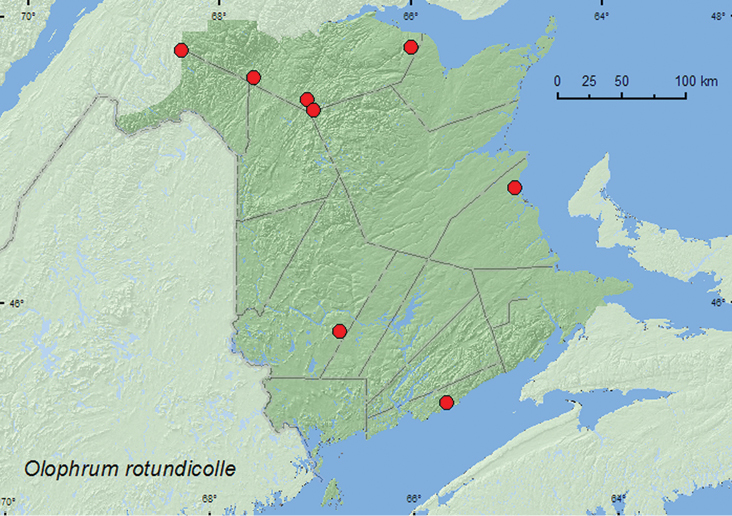
Collection localities in New Brunswick, Canada of *Olophrum rotundicolle*.

##### Collection and habitat data.

This Holarctic species has been collected from moss and *Carex* hummocks, along lake, stream, and bog margins, from floating debris on streams, and in moist *Salix* and *Alnus* spp. litter (Campbell 1983b). Most specimens from New Brunswick were collected from moss, sphagnum, and leaf litter in and near eastern white cedar swamps and in *Carex* marshes. Adults were collected by sifting moss and litter or treading *Carex* hummocks and vegetation into water. This species was collected during April, May, June, July, and August.

##### Distribution in Canada and Alaska.

AK, YT, NT, BC, AB, SK, MB, ON, QC, **NB**, NS, LB, NF (Campbell 1983b; CNC specimens).

#### Porrhodites inflatus

(Hatch, 1957)**

http://species-id.net/wiki/Porrhodites_inflatus

Map 10

##### Material examined.

**New Brunswick, York Co.** Charters Settlement, 45.8260°N, 66.7376°W, 29.XI.2004, R. P. Webster, mixed forest, on surface of puddle on forest trail after heavy rain (17, NBM, RWC).

**Map 10.**
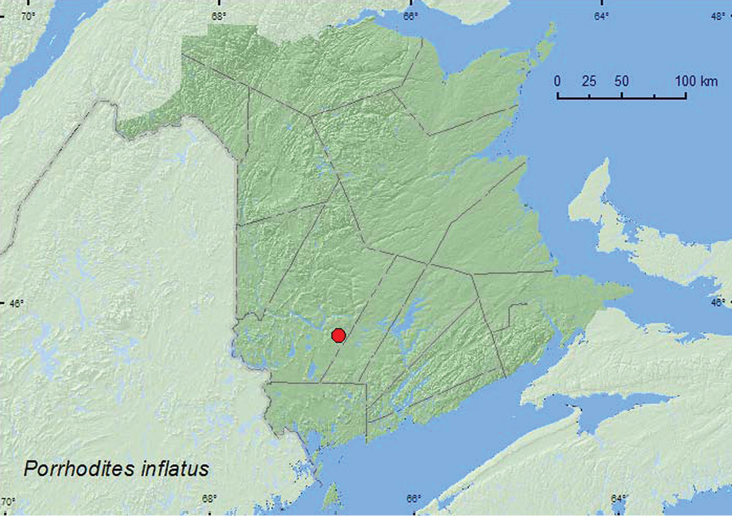
Collection localities in New Brunswick, Canada of *Porrhodites inflatus*.

##### Collection and habitat data.

This species is rarely collected, in part because it is primarily active in the late fall and early winter (Campbell 1984b). Adults have been found crawling on snow on a warm day in November and by sifting moss and plant debris in October and November (Campbell 1984b). The New Brunswick specimens were found floating on water on the surface of frozen puddles along a forest trail after a heavy rain the previous night that had melted a 10 cm deep snow cover. Presumably the adults were washed into the puddles by the heavy rain. The adults were collected in late November.

##### Distribution in Canada and Alaska.

BC, AB, ON, QC, **NB** (Campbell 1984b).

#### Trigonodemus striatus

LeConte, 1863

http://species-id.net/wiki/Trigonodemus_striatus

Map 11

##### Material examined.

**New Brunswick, Carleton Co.**, Meduxnekeag Valley Nature Preserve, 46.1897°N, 67.6710°W, 26.IX.2007, 12.IX.2008, R. P. Webster, mature mixed forest (with many white pine), on *Pholiota* species at base of dead black cherry and base of dead *Populus* species (6, RWC). **Charlotte Co.**, near New River, 45.2122°N, 66.6160°W, 22.IX.2006, R. P. Webster, (old growth) eastern white cedar swamp, in gilled mushroom (*Pholiota* sp. on log) (1, RWC). **Restigouche Co.**, Dionne Brook P.N.A., 47.9030°N, 68.3503°W, 19.IX.2011, R. P. Webster, old-growth northern hardwood forest, in gilled mushroom (1, RWC).

**Map 11.**
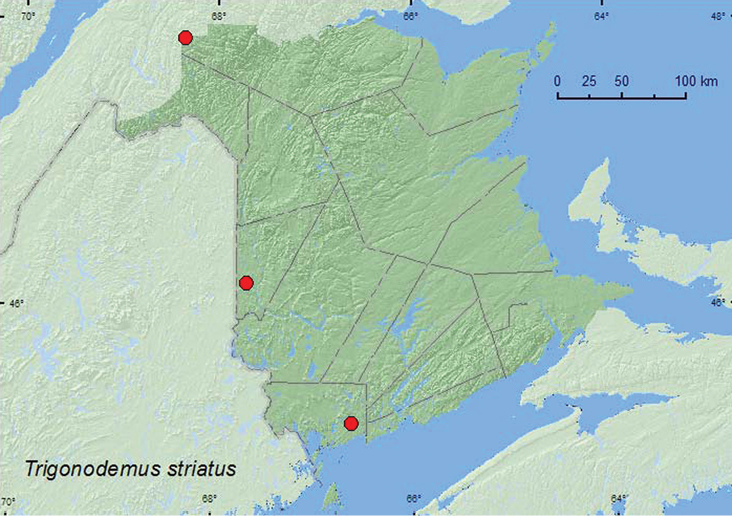
Collection localities in New Brunswick, Canada of *Trigonodemus striatus*.

##### Collection and habitat data.

This is a fungicolous species occurring in various species of mushrooms (including *Russula* sp.), typically those growing on rotting wood (Smetana 1996). Smetana (1996) reported that numerous specimens were captured in flight intercept traps. All records from Smetana (1996) were from September and October. In New Brunswick, adults were collected during September on *Pholiota* sp. at the base of a dead black cherry (*Prunus serotina* Ehrh.), a dead *Populus* sp., and on a log. One individual was collected from a gilled mushroom on the forest floor. Adults were found in a mature mixed forest, an eastern white cedar swamp, and in an old-growth northern hardwood forest.

##### Distribution in Canada and Alaska.

ON, QC, **NB**, NS (Smetana 1996).

### Tribe Coryphiini Jakobson, 1908

#### Coryphium nigrum

Campbell, 1978

http://species-id.net/wiki/Coryphium_nigrum

Map 12

##### Material examined. 

**New Brunswick, Albert Co.**, Caledonia Gorge P.N.A., at Canada Creek, 45.7808°N, 64.7775°W, 4.VII.2011, R. P. Webster, cold, clear, and shaded rocky brook in mixed forest, in saturated moss (1, NBM); Caledonia Gorge P.N.A. at Caledonia Creek, 45.7935°N, 64.7760°W, 1.VII.2011, R. P. Webster, shaded, rocky, cold, clear brook, splashing gravel (2, NBM). **Carleton Co.**, Meduxnekeag Valley Nature Preserve, 46.1895°N, 67.6704°W, 13.VI.2010, 18.VI.2010, R. P. Webster, hardwood forest, margin of cold shaded spring-fed brook, splashing gravel, sand and clay mix (4 ♂, 3 ♀, NBM, RWC). **Madawaska Co.**, Gagné Brook at First Lake, 47.6077°N, 68.2534°W, 23.VI.2010, M. Turgeon & R. Webster, northern hardwood forest, shaded brook among gravel on gravel bar, splashing and turning pebbles (1 ♂, 1 ♀, RWC); Jalbert Brook, 262 m elev., 47.6470°N, 68.3026°W, 23.VI.2010, R. P. Webster, old growth mixed forest, shaded brook, in gravel on gravel bar (3 ♂, 4 ♀, NBM, RWC).

**Map 12.**
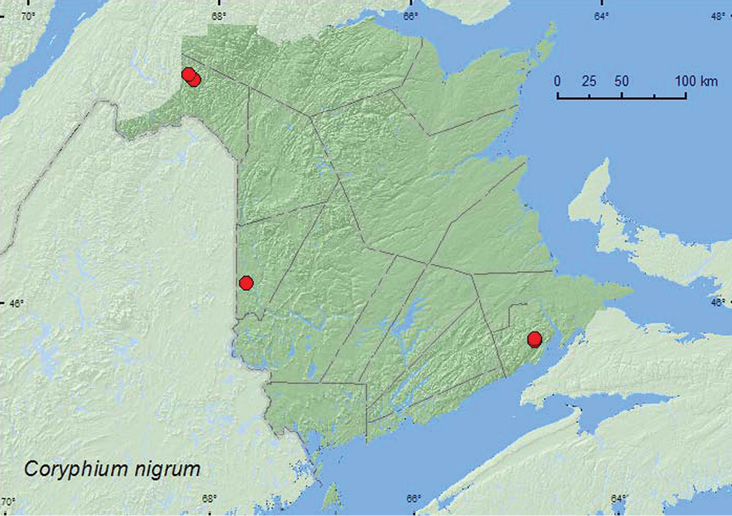
Collection localities in New Brunswick, Canada of *Coryphium nigrum*.

##### Collection and habitat data.

In New Brunswick, *Coryphium nigrum* was found on margins of heavily shaded brooks, usually on a gravel, sand, and clay mix. One teneral individual was collected from saturated moss on a rock in a brook. Adults were collected either by turning pebbles and gravel or more easily by lightly splashing the brook margin. Adults were collected after they moved to the tops of the pebbles, but were often difficult to see due to the low light levels of the habitat. Nothing was previously known about the biology of this species other than that adults were collected in late March through May and in September (Campbell 1978). Campbell (1978) suggested that adults were probably most active during spring or even late winter like other members of the tribe. In New Brunswick, adults were collected from mid to late June and early July, and were common at this time.

##### Distribution in Canada and Alaska. 

QC, **NB**, NS (Campbell and Davies 1991; CNC specimens).

### Subfamily Micropeplinae Leach, 1815

The Micropeplinae were reviewed by Campbell (1968). Campbell (1973b, 1978b) later described three new species and provided additional distributional and habitat data for other species. Adults occur in forest duff or detritus, in or near swamps and bogs, or in more restricted habitats such as bird and mammal nests, but are rarely collected (Campbell 1968). *Micropeplus browni* Campbell and *Micropeplus laticollis* Mäklin are newly reported for New Brunswick and the Maritime provinces.

#### Micropeplus browni

Campbell, 1968**

http://species-id.net/wiki/Micropeplus_browni

Map 13

##### Material examined.

**New Brunswick,** Charters Settlement, 45.8395°N, 66.7391°W, 10.V.2007, R. P. Webster, mixed forest, u.v. light (1, RWC).

**Map 13.**
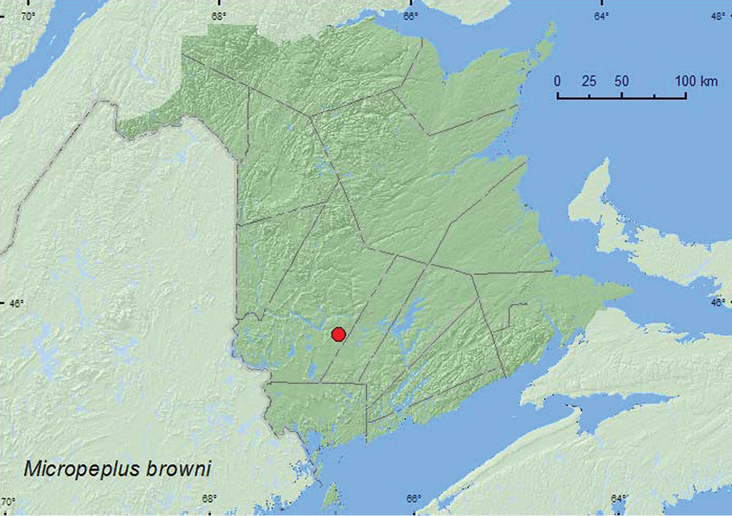
Collection localities in New Brunswick, Canada of *Micropeplus browni*

##### Collection and habitat data.

Campbell (1968) reported that most specimens of this species were collected from beaver (*Castor canadensis* Kuhl) lodges, but three individuals were taken from an animal nest under a log. The specimen from New Brunswick was collected during May at an ultraviolet light near a mixed forest.

##### Distribution in Canada and Alaska.

ON, QC, **NB** (Campbell 1968; Campbell and Davies 1991)

#### Micropeplus laticollis

Mäklin, 1853**

http://species-id.net/wiki/Micropeplus_laticollis

Map 14

##### Material examined.

**New Brunswick, Restigouche Co.**, Dionne Brook P.N.A., 47.9064°N, 68.3441°W, 31.V–15.VI.2011, 15–27.VI.2011, 27.VI–14.VII.2011, 9–23.VIII.2011, M. Roy & V. Webster, old-growth white spruce and balsam fir forest, Lindgren funnel traps (17, AFC, NBM, RWC).

**Map 14.**
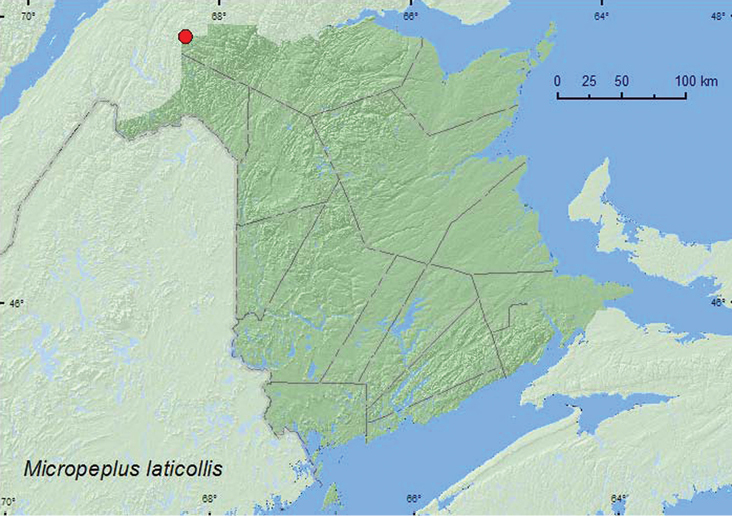
Collection localities in New Brunswick, Canada of *Micropeplus laticollis*.

##### Collection and habitat data.

Adults have been taken in Berlese samples of conifer (various species) duff, a red squirrel (*Tamiasciurus hudsonicus* Erxl.) midden, and nests of Canada Jay (*Perisoreus canadensis captitalis* Baird) (Campbell 1968). A number of specimens were collected from *Salix*, *Alnus*, and other deciduous litter near streams (Campbell 1973b). Adults from New Brunswick were captured in Lindgren funnel traps deployed in an old-growth white spruce and balsam fir forest (boreal forest). Adults were captured during June, July, and August.

##### Distribution in Canada and Alaska.

AK, YK, BC, AB, SK**, MB,** ON, QC, **NB** (Campbell 1968, 1973b, 1979; CNC specimens).

### Subfamily Phloeocharinae Erichson, 1839

Two genera and species in this subfamily, the eastern *Charhyphus picipennis* (LeConte) and the western *Vicelva vandykei* (Hatch), were previously known from Canada (Campbell and Davies 1991; Newton et al. 2000). Majka and Klimaszewski (2004) later reported the adventive *Phloeocharis subtilissma* Mannerheim to the fauna of Nova Scotia and North America. *Charhyphus picipennis* is reported here for the first time for New Brunswick and represents the first record of this subfamily for the province.

#### Charhyphus picipennis

(LeConte, 1863)

http://species-id.net/wiki/Charhyphus_picipennis

Map 15

##### Material examined.

**New Brunswick, Carleton Co.**, Jackson Falls, Bell Forest, 46.2200°N, 67.7231°W, 12–19.VI.2008, R. P. Webster, mature hardwood forest with some conifers, Lindgren funnel traps (2, AFC, RWC); same locality, forest type and collector but 28.IV^=9.V.2009, 9–14.V.2009, Lindgren funnel traps (2, AFC). **Queens Co.**, W of Jemseg near “Trout Creek”, 45.8227°N, 66.1240°W, 3.VI.2007, R. P. Webster, silver maple forest, under tight bark of *Ulmus americana* (1 RWC); Grand Lake near Scotchtown, 45.8762°N, 66.1817°W, 30.IV.2006, R. P. Webster, red oak forest near lake, under bark of red oak log (1, RWC); Cranberry Lake P.N.A, 46.1125°N, 65.6075°W, 24.IV–5.V.2009, 27.V–5.VI.2009, R. Webster & M.-A. Giguère, mature red oak forest, Lindgren funnel traps (3, AFC). **Restigouche Co.**, Dionne Brook P.N.A., 47.9030°N, 68.3503°W, 30.V–15.VI.2011, M. Roy & V. Webster, old-growth northern hardwood forest, Lindgren funnel trap (1, NBM). **York Co.**, Charters Settlement, 45.8395°N, 66.7391°W, 30.V.2007, R. P. Webster, mixed forest, under tight bark of dead standing balsam fir (1, RWC); 15 km W of Tracy off Rt. 645, 45.6848°N, 66.8821°W, 4–11.V.2009, 19–25.V.2009, 6–15.VI.2009, R. Webster & M.-A. Giguère, old red pine forest, Lindgren funnel traps (3, AFC); 14 km WSW of Tracy, S of Rt. 645, 45.6741°N, 66.8661°W, 28.IV–10.V.2010, R. Webster & C. MacKay, old mixed forest with red and white spruce, red and white pine, balsam fir, eastern white cedar, red maple, and *Populus* sp., Lindgren funnel trap (1, AFC).

**Map 15.**
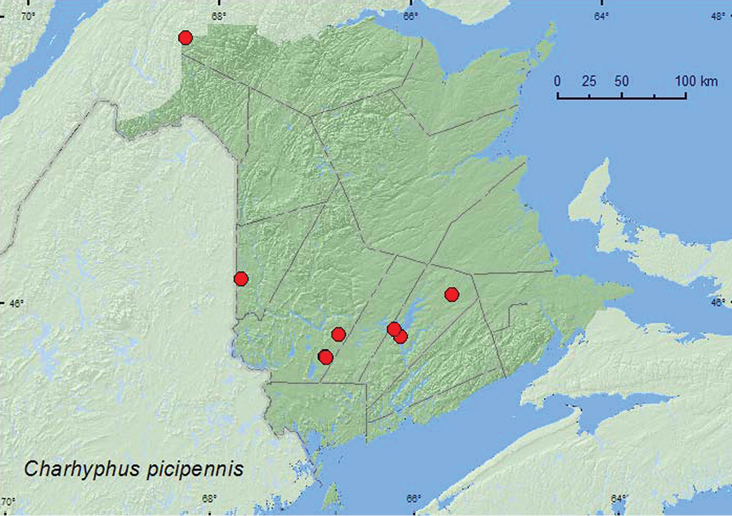
Collection localities in New Brunswick, Canada of *Charhyphus picipennis*.

##### Collection and habitat data.

Members of this genus typically occur under bark of hardwoods (Newton et al. 2000). *Charhyphus picipennis* was frequently collected in Lindgren funnel traps in various forest types in New Brunswick and was found under tight bark of American elm (*Ulmus americana* L.) and red oak. Adults were collected during April, May, and June.

##### Distribution in Canada and Alaska. 

ON, QC, **NB**, NS (Campbell and Davies 1991).

### Subfamily Olisthaerinae Thomson, 1858

The two Holarctic species, *Olisthaerus megacephalus* (Zetterstedt) and *Olisthaerus substriatus* (Paykull), are the only members of this subfamily recorded from Canada and North America (Campbell and Davies 1991; Newton et al. 2000). Both species live under bark of conifers (Newton et al. 2000).

#### Olisthaerus substriatus

(Paykull, 1790)**

http://species-id.net/wiki/Olisthaerus_substriatus

Map 16

##### Material examined.

**New Brunswick, Restigouche Co.**, Little Tobique River near Red Brook, 47.4462°N, 67.0689°W, 24.V.2007, R. P. Webster, old growth eastern white cedar swamp, under bark of large fallen spruce (9, NBM, RWC); MacFarlane Brook P.N.A, 47.6018°N, 67.6263°W, 25.V.2007, R. P. Webster, old growth eastern white cedar swamp, under bark of large fallen spruce (1, RWC); Berry Brook P.N.A, 47.8134°N, 66.7579°W, 26.V.2007, R. P. Webster, old growth eastern white cedar swamp, under bark of large fallen spruce (2 ♂, NBM, RWC). **York Co.**, 15 km W of Tracy off Rt. 645, 45.6837°N, 66.8809°W, 10.VI.2007, R. P. Webster, old red pine forest, underside of red pine log under bark (1, RWC); 15 km W of Tracy off Rt. 645, 45.6848°N, 66.8821°W, 29.VII–4.VIII.2009, R. Webster & M.-A. Giguère, old red pine forest, Lindgren funnel trap (1, AFC); same locality and habitat data but 10–26.V.2010, R. Webster & C. MacKay, Lindgren funnel trap (1, AFC).

**Map 16.**
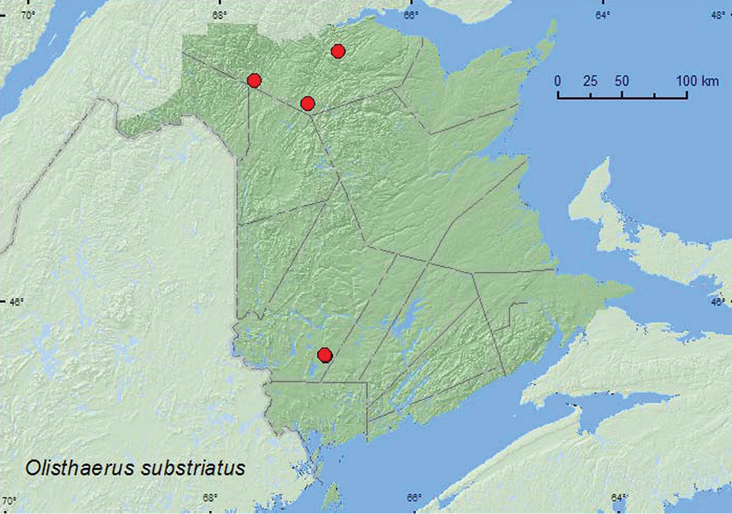
Collection localities in New Brunswick, Canada of *Olisthaerus substriatus*.

##### Collection and habitat data.

This species typically occurs under bark of dead conifers (Newton et al. 2000), the same habitat from which most New Brunswick specimens were collected. Adults were collected from under bark of large fallen spruce and under bark on the underside of a large red pine log. Two adults were captured in Lindgren funnel traps in an old-growth red pine forest. Adults were collected during May, June, July, and August.

##### Distribution in Canada and Alaska.

YT, NT, AB, SK, ON, QC, **NB** (Campbell and Davies 1991; CNC specimens). This species and subfamily is newly reported for the Maritime provinces.

### Subfamily Habrocerinae Mulsant & Rey, 1876

Assing and Wunderle (1995) reviewed the Habrocerinae of the world. *Habrocerus capillaricornis* (Gravenhorst), *Habrocerus schwarzi* Horn, and *Habrocerus magnus* LeConte are the only members of this subfamily in Canada and North America (Campbell and Davies 1991; Newton et al. 2000). Although Assing and Wunderle (1995) excluded *Habrocerus magnus* from the Habrocerinae, they did not place it in any other subfamily. Newton et al. (2000) suggested that this species may belong in the Olisthaerinae based on larval characters. We retain *Habrocerus magnus* in the Habrocerinae and the Genus *Habrocerus* pending formal placement elsewhere. Most members of the Habrocerinae occur in litter, wood debris, and fungi (Assing and Wunderle 1995; Newton et al. 2000). No species of Habrocerinae were previously known from New Brunswick. Here, we report the first records of this subfamily from the province.

#### Habrocerus capillaricornis

(Gravenhorst, 1806)

http://species-id.net/wiki/Habrocerus_capillaricornis

Map 17

##### Material examined.

**New Brunswick, Carleton Co.**, Jackson Falls, Bell Forest, 46.2200°N, 67.7231°W, 12.VII.2006, R. P. Webster, mature hardwood forest, u.v. light (1, RWC). **York Co.**, Charters Settlement, 45.8188°N, 66.7460°W, 15.VIII.2004, R. P. Webster, mixed forest, in decaying fungi (1, NBM); same locality but 45.8340°N, 66.7450°W, 27.IV.2005, R. P. Webster, mixed forest, in woodpile, under bark of spruce (1, NBM); same locality but 45.8395°N, 66.7391°W, 5.VIII.2006, 22.VIII.2006, R. P. Webster, mixed forest, in pile of decaying leaves (7 (many other individuals were observed), RWC); New Maryland, U.N.B. Woodlot, 45.9116°N, 66.6698°W, 26.V.2008, R. Webster, G. Forbes, & M.-A. Giguère, abandoned beaver lodge occupied by muskrats, in wall of lodge (1, NBM).

**Map 17.**
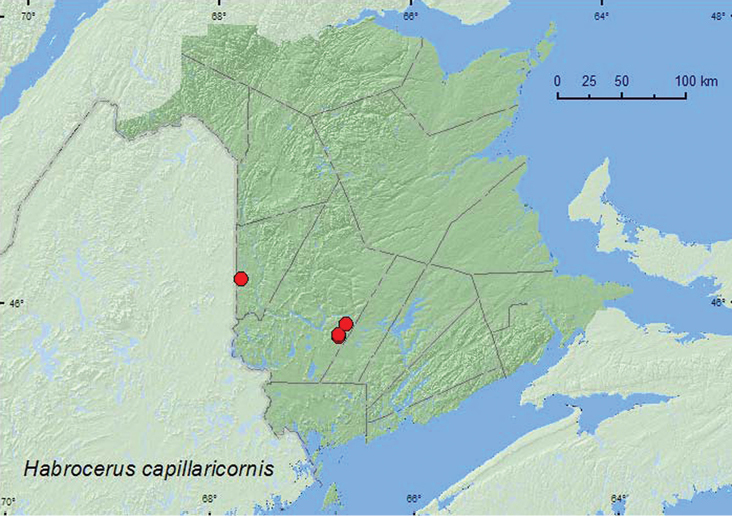
Collection localities in New Brunswick, Canada of *Habrocerus capillaricornis*.

##### Collection and habitat data.

*Habrocerus capillaricornis* has been reported from forested areas under bark, in litter, in fungi (Assing and Wunderle 1995) and from compost (Brunke et al. 2011). In New Brunswick, this adventive species was common among decaying leaves in a pile of leaves made the previous year. Other adults were observed among a pile of decaying corncobs and cornhusks nearby and from compost (Webster, unpublished data). Adults were also found under bark of spruce in a woodpile, in decaying fungi, in the wall of a beaver lodge and at a black-light trap. Adults were collected during April, May, July, and August.

##### Distribution in Canada and Alaska.

BC, MB, ON, QC, **NB**, NS, NF (Campbell and Davies 1991; Majka and Klimaszewski 2008; CNC specimens).

#### Habrocerus schwarzi

Horn, 1877**

http://species-id.net/wiki/Habrocerus_schwarzi

Map 18

##### Material examined.

**New Brunswick, Restigouche Co.**, Jacquet River Gorge P.N.A., 47.8160°N, 66.0083°W, 14.VIII.2010, R. P. Webster, old eastern white cedar forest, in decaying mushrooms (2 ♂, NBM, RWC). **York Co.**, Canterbury, near Browns Mountain Fen, 45.8954°N, 67.6307°W, 7.IX.2007, R. P. Webster, mixed forest along forest trail, in decaying gilled mushrooms (1 ♂, 2 ♀, RWC).

**Map 18.**
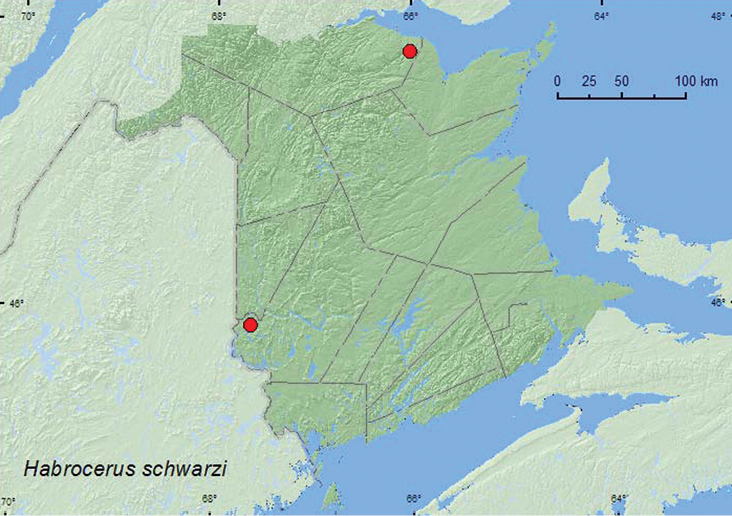
Collection localities in New Brunswick, Canada of *Habrocerus schwarzi*.

##### Collection and habitat data.

Assing and Wunderle (1995) reported that this species was most often collected from well-decayed fungi, but was also found in moose (*Alces alces* L.) and grouse (*Bonasa umbellus* L.) dung and leaf litter. Paquin and Dupérré (2002) captured large numbers of this species in pitfall traps deployed in the southern boreal forest of Quebec. The specimens from New Brunswick were found in decaying gilled mushrooms in a mature mixed forest and an old eastern white cedar forest. Adults were collected during August and September.

##### Distribution in Canada and Alaska.

AB, MB, ON, QC, **NB** (Campbell and Davies 1991).

#### Habrocerus magnus

LeConte, 1878**

http://species-id.net/wiki/Habrocerus_magnus

Map 19

##### Material examined.

**New Brunswick, Charlotte Co.**, near Little Pocologan River, 45.1731°N, 66.6141°W, 7.V.2007, R. P. Webster, clear-cut, under bark of large *Pinus strobus* (white pine) log (1, RWC). **York Co.**, 15 km W of Tracy off Rt. 645, 45.6837°N, 66.8809°W, 16.VI.2007, R. P. Webster, small clear-cut, under bark of red pine stump (1, RWC); same locality, collector but 45.6848°N, 66.8821°W, 27.VIII.2008, R. P. Webster, old red pine forest, under bark of large standing dead white pine (4, RWC).

**Map 19.**
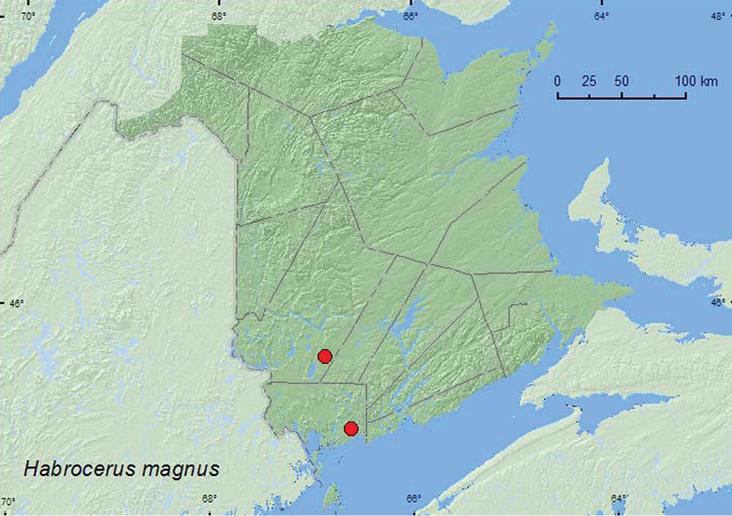
Collection localities in New Brunswick, Canada of *Habrocerus magnus*.

##### Collection and habitat data.

Brunke et al. (2011) noted that almost nothing was known about this rarely collected species but mentioned that it shared morphological features with other Staphylinidae living in subcortical habitats. Most adults of*Habrocerus magnus* from New Brunswick were found under somewhat loose bark of white pine (*Pinus strobus* L.) (large logs and a large, dead, standing tree). One individual was collected from under bark of a large red pine stump. These data suggest that this species lives under bark of large dead conifers. Adults were collected during May, June, and August.

##### Distribution in Canada and Alaska.

MB, ON, QC, **NB**, NF (Campbell and Davies 1991; CNC specimens).

## Supplementary Material

XML Treatment for Hapalaraea hamata

XML Treatment for Phloeonomus laesicollis

XML Treatment for Acidota quadrata

XML Treatment for Eucnecosum brunnescens

XML Treatment for Eucnecosum tenue

XML Treatment for Geodromicus strictus

XML Treatment for Microedus austinianus

XML Treatment for Olophrum obtectum

XML Treatment for Olophrum rotundicolle

XML Treatment for Porrhodites inflatus

XML Treatment for Trigonodemus striatus

XML Treatment for Coryphium nigrum

XML Treatment for Micropeplus browni

XML Treatment for Micropeplus laticollis

XML Treatment for Charhyphus picipennis

XML Treatment for Olisthaerus substriatus

XML Treatment for Habrocerus capillaricornis

XML Treatment for Habrocerus schwarzi

XML Treatment for Habrocerus magnus
